# PBI-05204, a supercritical CO_2_ extract of *Nerium oleander*, inhibits growth of human pancreatic cancer via targeting the PI3K/mTOR pathway

**DOI:** 10.1007/s10637-014-0190-6

**Published:** 2014-12-06

**Authors:** Yong Pan, Patrea Rhea, Lin Tan, Carrie Cartwright, Ho-Jeong Lee, Murali K. Ravoori, Crandell Addington, Mihai Gagea, Vikas Kundra, Sun-Jin Kim, Robert A. Newman, Peiying Yang

**Affiliations:** 1 Department of General Oncology, The University of Texas MD Anderson Cancer Center, 1515 Holcombe Blvd., Unit 0462, Houston, TX 77030 USA; 2 Department of Cancer Biology, The University of Texas, MD Anderson Cancer Center, 1515 Holcombe Blvd., Unit 0173, Houston, TX 77030 USA; 3 Department of Cancer Systems Imaging, The University of Texas, MD Anderson Cancer Center, 1515 Holcombe Blvd., Unit 1907, Houston, TX 77030 USA; 4 Phoenix Biotechnology, 8626 Tesoro Dr., San Antonio, TX 78217 USA; 5 Department of Veterinary Medicine & Surgery, The University of Texas, MD Anderson Cancer Center, 1515 Holcombe Blvd., Unit 0063, Houston, TX 77030 USA; 6 Department of Diagnostic Radiology, The University of Texas, MD Anderson Cancer Center, 1515 Holcombe Blvd., Unit 1473, Houston, TX 77030 USA; 7 Department of Experimental Therapeutics, The University of Texas, MD Anderson Cancer Center, 1515 Holcombe Blvd., Houston, TX 77030 USA

**Keywords:** *Nerium oleander*, PBI-05204, Panc-1, Orthotopic, PI3K/mTOR

## Abstract

*Introduction* Oleandrin, a cardiac glycoside, exerts strong anti-proliferative activity against various human malignancies in in vitro cells. Here, we report the antitumor efficacy of PBI-05204, a supercritical C0_2_ extract of *Nerium oleander* containing oleandrin, in a human pancreatic cancer Panc-1 orthotopic model. *Results* While all the control mice exhibited tumors by the end of treatment, only 2 of 8 mice (25 %) treated for 6 weeks with PBI-05204 (40 mg/kg) showed dissectible tumor at the end of the treatment period. The average tumor weight (222.9 ± 116.9 mg) in mice treated with PBI-05204 (20 mg/kg) was significantly reduced from that in controls (920.0 ± 430.0 mg) (*p* < 0.05). Histopathologic examination of serial sections from each pancreas with no dissectible tumor in the PBI-05204 (40 mg/kg) treated group showed that the pancreatic tissues of 5/6 mice were normal while the remaining mouse had a tumor the largest diameter of which was less than 2.3 mm. In contrast, while gemcitabine alone did not significantly reduce tumor growth, PBI-05204 markedly enhanced the antitumor efficacy of gemcitabine in this particular model. Ki-67 staining was reduced in pancreatic tumors from mice treated with PBI-05204 (20 mg/kg) compared to that of control, suggesting that PBI-05204 inhibited the proliferation of the Panc-1 tumor cells. PBI-05204 suppressed expression of pAkt, pS6, and p4EPB1 in a concentration-dependent manner in both Panc-1 tumor tissues and human pancreatic cancer cell lines, implying that this novel botanical drug exerts its potent antitumor activity, at least in part, through down-regulation of PI3k/Akt and mTOR pathways.

## Introduction

Pancreatic cancer is the fourth most common cause of cancer deaths in Western society and is one of the leading causes of cancer deaths worldwide. In 2014, an estimated 46,420 new cases will be diagnosed, and an estimated 39,590 people in the United States will die from this disease [1]. Despite progress made in our understanding of the genetic causes of invasiveness of this disease and efforts to improve the treatment of pancreatic cancer with various therapeutic regimens, the survival figures for pancreatic cancer have not changed significantly over the past four decades [2]. Stage-for-stage, cancer of the pancreas has the shortest median survival time of all cancer types. Therefore, there is a critical need to not only understand the nature and molecular activity of this deadly disease, but to also develop effective treatments as well. Finding drugs that can specifically target tumor cells, yet not harm normal cells, while causing minimal systemic side effects has become a major focus of today’s research.

Cardiac glycosides are a class of compounds used to treat congestive heart failure and do so by increasing myocardial contractile force [3]. Oleandrin is a cardiac glycoside derived from *Nerium oleander* that has been used extensively over the years in Russia and China for this purpose. In addition, over the past century, oleander has been used in traditional remedies and in commercial preparations in various parts of the world for a multitude of ailments, including inflammation, parasitic and bacterial infections, cardiac abnormalities, ringworm, skin diseases, swelling, vomiting, warts, and excessive weight gain. Today, much of the focus on oleander research has centered on anticancer and antiviral activities [4–8]. In vitro, oleandrin has been shown to block ceramide-induced NF-kappa B activation in a number of model biological systems including human epithelial and lymphoid cells, insect, and murine macrophage cells [9]. Other studies have suggested that oleandrin and PBI-05204 exhibit neuroprotective effects against neural tissues damaged by oxygen and glucose deprivation in ischemic stroke [10]. Cytotoxicity tests have revealed a broad range of anti-proliferative efficacy against various tumor cell lines including HeLa and A498 [11] as well as inhibition of FGF-2 export in vitro from PC3 and DU145 prostate cancer cells in a concentration- and time-dependent fashion which together contribute to the antitumor activity of this novel treatment for cancer [12]. Preclinical and retrospective patient data suggest that cardiac glycosides (e.g. digoxin, digitoxin, ouabain and oleandrin) affect the growth of various cancers including breast, lung, prostate and leukemia [13]. For example, oleandrin can inhibit the P-gp pump in HL60 and K562 leukemia cell lines [14]. The relative sensitivity of cancer cells to oleandrin treatment was shown to be species-specific [15]; our laboratory has also shown that these compounds induce selective cell death in certain human malignant cells but not murine tumor cells or normal human cells, a phenomenon likely mediated through both expression and distribution of the α3 isoform of Na, K-ATPase the target to which CGs bind [4, 16]. Although much has been published on how oleandrin mediates its biological activity in vitro, knowledge on how it inhibits tumor growth in the human pancreatic cancer mouse orthotopic model remains limited.

In the present study, we evaluated the antitumor efficacy of PBI-05204, a modified supercritical CO_2_ extract of *Nerium oleander*, with or without gemcitabine, in a human pancreatic cancer Panc-1 mouse orthotopic model in an attempt to understand its relative efficacy and underlying pharmacologic mechanisms. The data obtained suggest that PBI-05204 exerts potent antitumor efficacy, superior to that of gemcitabine alone, a standard therapy for pancreatic cancer.

## Materials and methods

### Chemical, reagents and antibodies

Phospho-AKT, phospho-S6, and phospho-4EBP1were purchased from Cell Signaling Technology (Beverly, MA). Ki-67 was purchased from Thermoscientific (Waltham, MA) and β-Actin was purchased from Sigma (St. Louis, MO). RNase A and 3-(4,5-dimethylthiazol-2-yl)-2,5-diphenyltetrazolium bromide (MTT) were also purchased from Sigma Chemical, Co. PBI-05204, a supercritical CO_2_ extract, was prepared from the leaves of organically grown *Nerium oleander* plants by Flavex Naturextrakte (Rehlingen, Germany).

#### Cell lines and cell culture

Human pancreatic cancer Panc-1 and Capan-II cells were obtained from the American Type Culture Collection (Manassas, VA), maintained in a humidified atmosphere containing 5 % CO_2_ at 37 °C and were routinely cultured in DMEM tissue culture medium (Invitrogen Corp., Grand Island, NY) supplemented with 10 % heat inactivated fetal bovine serum ([FBS] Hyclone Laboratories Inc., Logan, UT), 50 IU/ml penicillin and 50 μg/ml streptomycin, and 2 mM L-glutamine from GIBCO (Invitrogen). All cell lines were authenticated via microscopic morphology check and DNA characterization.

#### Animals and study design

All animal study protocols were approved by the Animal Care and Use Committee of the University of Texas, M. D. Anderson Cancer Center. Female balb/c *nu*/*nu* 6–8 weeks old mice were supplied by Experimental Radiation Oncology, M. D. Anderson Cancer Center, TX and were allowed to acclimatize for 3 days prior to study initiation. Mice were fed AIN76 diet (Harlan, Livermore, CA) and water *ad libitum*. For development of the orthotopic model, mice were anesthetized with Ketamine: Xylazine cocktail (150 mgkg: 7.5 mg/kg IP) followed with an aseptic scrub process. The abdominal skin and muscle were incised just off the midline and directly above the pancreas to allow visualization of the pancreatic lobes. The pancreas was gently retracted and positioned to allow for direct injection of a 50 μL bolus containing 1 × 10^6^ Panc-1 cells/HBSS using a 1 cc syringe with a 30 gauge needle. The pancreas was placed back within the abdominal cavity, and the muscle and skin layers were closed with surgical sutures and staples. Analgesia (Buprenorphine 0.05 mg/kg SC) was given twice immediately following surgery and 12 h later. Fourteen days after injection, all animals that received orthotopic tumor cells inoculation were imaged using an MR Scanner in the Small Animal Imaging Facility (SAIF, MD Anderson Cancer Center, TX). MR studies were performed on a 7T scanner (Bruker Biospec, Bruker Biospin, Billerica, MA) using a 60-mm gradient insert and a volume resonator with a 35 mm inner diameter. Animals were anesthetized and placed head first and prone on a positioning sled. Orthogonal 3-plane scout scans were initially acquired for animal positioning. MR images were acquired using a T_2_-weighted rapid acquisition with relaxation enhancement (RARE) pluse sequence repetition time (TR) =2453.85 ms; echo time (TE) =38 ms; flip angle (FA) =180°; field of view (FOV) =4×3×3 cm^3^; slice thickness =0.75 mm, image matrix =256 × 192; number of signal averages =3). Only mice with positive tumor imaging were randomized to seven different treatment groups including vehicle control (25 % DMSO: 75 % PEG400), PBI-05204 (10, 20 and 40 mg/kg), Gemcitabine (40 mg/kg) and PBI-05204/Gemcitabine (10 and 20 mg/kg/40 mg/kg). PBI-05204 was administered to mice via an oral gavage daily while gemcitabine was given by i. p. injection three times per week for 6 weeks. Animals were weighed weekly and observed daily. MR Imaging was performed before treatment and 6 weeks after initial treatment with PBI-05024. At the end of treatment, blood was collected by cardiac puncture and detected pancreatic tumor was removed, weighed and measured. Tumor tissues were either formalin fixed or flash frozen in liquid nitrogen and then stored at −80 °C for future analysis. Tumor burden was defined as total tumor volume multiplied by the number of detectable tumors.

#### Histopathology and immunohistochemistry

Tumor tissues, pancreas and mesenterium were collected from PBI-05204 treated mice, fixed in neutral buffered formalin, and paraffin processed for histopathological examination (H&E stained sections) or biomarker identification by immunohistochemistry staining. For pancreas without macroscopically visible tumors, the entire pancreas and mesenteric tissue were serially sectioned and stained with H&E every 50 μm, and then were examined microscopically. For IHC staining, 6 μm thick sections were mounted on glass slides, and antigens were unmasked using antigen retrieval. After washing in Tris buffer solution, sections were incubated in a methanol peroxide solution followed by blocking with normal goat serum and BSA. Sections were incubated with monoclonal antibodies in a humidified chamber overnight at 4 °C. After washing with Tris buffer solution, an appropriate secondary antibody was added and samples were incubated at room temperature for 45 min. For antibody visualization, sections were incubated with ABC (Vector Laboratories, Burlingame, CA) followed by DAB substrate and finally counterstained with Mayer’s haematoxylin, dehydrated and mounted in Permount. Immunostaining was assessed with an EVOS light microscope (AMG, WA).

#### Determination of mouse plasma oleandrin content

To prepare samples for determination of oleandrin content, 0.2 mL of plasma was diluted with 0.8 mL distilled water containing 100 ng/ml of cinobufatalin (internal standard) and then applied to a preconditioned Waters Oasis HLB solid phase extraction cartridge (1 cc 30 mg, Waters Corp., Milford, MA). The cartridge was then washed with 2 mL of water and oleandrin was eluted with 1 mL of ethyl acetate followed by 1 mL of methanol, after which the eluent was collected and evaporated. The dried sample was then reconstituted in 200 μl methanol: 0.2 % formic acid (1:1) prior to analysis by LC/MS/MS.

Reverse-phase HPLC electrospray ionization MS was performed using an Agilent 6460 triple quadruple tandem mass spectrometer equipped with an Agilent 1200 HP binary pump high-pressure LC inlet. Oleandrin was separated using a Kinetex C18 2.6 μ (2 × 100 mm) LC column (Phenomenex, Torrence, CA). The mobile phase consisted of 0.2 % formic acid (aqueous), and methanol; the flow rate was 200 μl/min and a column temperature was maintained at 35 °C. The sample injection volume was 10 μl. The mass spectrometer was operated in the electrospray positive ion mode with a source temperature of 250°. The collision energy was 15 V for oleandrin and 10 V for the internal standard. The multiple-reaction monitoring (MRM) of the transition ions (M/Z) 577.3 > 373 for oleandrin and 459.2 > 363.1 for cinobufatalin were used for identification and quantification of oleandrin.

#### Cytotoxicity determination

Cells were grown at a density of 1 × 10^4^ cells/well in their relevant media. After a 24 h incubation period, cells were treated with various concentrations of PBI-05204 (0.2 to 100.0 μg/ml). After an additional 72 h, inhibition of cellular proliferation was assessed by MTT assay [17]. Absorbance was read at a wavelength of 570 nm and a reference wavelength of 650 nm using a V-Max Micro-plate Reader by Molecular Devices, Inc. (Sunnyvale, CA).

#### Cell cycle

Cells were treated with PBI-05204 as previously described. After 48 h treatment, cells were fixed with 70 % ETOH for at least 24 h. They were then washed with PBS, resuspended in PBS with PI (2 mg/ml) and RNase A solution (10 mg/ml), and measured using a flow cytometer/cell sorter.

#### Western blotting

PANC-1 cells (1 × 10^6^) were treated in serum-free conditions with 0.125, 0.25, 0.5 and 1 *u*g/ml PBI-05204 for 24 h. Cells were washed with cold phosphate buffered saline (PBS) and scraped with a lysis buffer (Invitrogen, Carlsbad, CA) with protease inhibitor cocktail (Sigma, Inc., St. Louis, MO) as an additive. Lysates were then sonicated on ice for 3 min., incubated for 10 min. and centrifuged at (14,000 g) for 10 min. at 4 °C. Protein levels were quantified via the BioRad Dc protein assay (BioRad, Inc., Hercules, CA). Equal levels of protein (50 μg) were fractionated on precast gels (BioRad) and then transferred on polyvinylidene diflouride membranes according to standard methods. Following a 1 h incubation in 5 % nonfat dry milk blocking buffer prepared in tris-buffered saline with 0.1 % tween 20 (TBS-T), membranes were probed with primary antibodies diluted 1:1000 in blocking buffer. Protein bands were visualized via chemiluminesence using the ECL + detection kit and hyper-film (Amersham Biosciences, Piscataway, NJ). Equal loading of samples was assessed by Western blotting for relative β-Actin content.

#### Statistical analysis

Student’s *t*-test or Mann-Whitney U test was used to determine the statistical differences between various experimental groups; a value of *P* ≤ 0.05 was considered to be significant.

## Results

### PBI-05204 substantially inhibited the proliferation of Panc-1 mouse orthotopic tumors

**Fig. 1:**
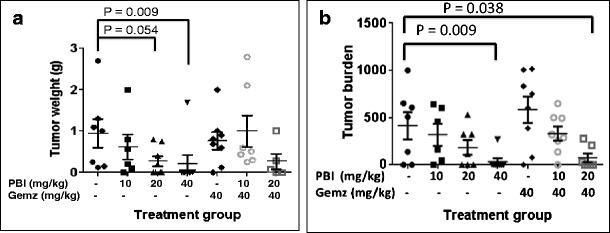
Tumor weight (**a**) and tumor burden (**b**) of human pancreatic cancer Panc-1 mouse orthotopic model treated with PBI-05204, gemcitabine (Gemz) or a combination of PBI-05204 and gemcitabine for 6 weeks. PBI-05204 but not gemcitabine significantly inhibited the growth of Panc-1 tumors. *Data* are presented as Mean ± SD (*n* = 7 for the control and 8 for each treatment group). *P* < 0.05 is the level of statistical significance

**Fig. 2:**
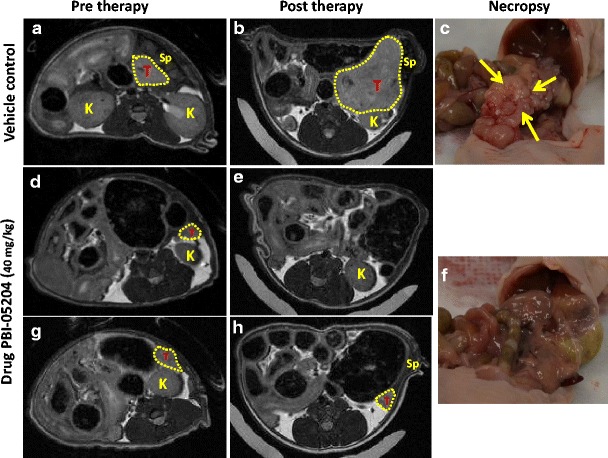
In vivo MR imaging shows that PBI-05204 reduces the growth of established Panc-1 tumors. Representative, axial MR (**a**, **b**, **d**, **e**, **g** and **h**) and coronal necropsy (**c** and **f**) *images* of nude mice (representative control and treatment group mice are shown). Treatment with PBI-05204 at a dose of 40 mg/kg daily via an oral gavage or vehicle was begun on day 14. MR imaging using a T_2_-weighted rapid acquisition with relaxation enhancement (RARE) pulse sequence was done 2 and 8 weeks after Panc-1 inoculation. T, pancreatic tumor. K, kidney. Sp, spleen. *Yellow arrows* indicate pancreatic tumor tissue in vehicle treated Panc-1 tumor bearing mice (**c**) whereas an example PBI-05204 (40 mg/kg) treated mouse showed normal looking pancreas (**f**)

To investigate the efficacy of PBI-05204 alone or in combination with a standard of care therapeutic agent for treatment of pancreatic cancer orthotopic tumors, mice bearing human pancreatic cancer Panc-1 cells were treated with PBI-05204 or gemcitabine individually or with a combination of both agents. PBI-05204 markedly and dose dependently inhibited the growth of Panc-1 tumor as evidenced both by a reduction of tumor weight (Fig. 1a) and tumor burden (Fig. 1b) while gemcitabine alone did not show any significant antitumor effect. The measurable tumor incidence was dose dependently reduced in PBI-05204 treated mice compared to vehicle treated group. The average tumor weight (222.9 ± 116.9 mg) in mice treated with PBI-05204 (20 mg/kg) was significantly reduced from that in controls (920.0 ± 430.0 mg) (*p* < 0.05) (Fig. 1a). This represented an average 75.8 % decline in tumor weight. The reduction of tumor growth by PBI-05204 (40 mg/kg) was remarkable and significant; all control mice exhibited dissectible tumors by the end of the treatment period while only 2 out of 8 mice (25 %) treated for 6 weeks with PBI-05204 (40 mg/kg) showed measureable pancreatic disease (*p* < 0.009, *n* = 8). The tumor sizes in both control and PBI-05204 (40 mg/kg) groups were similar before treatment (Fig. 2a, d and g). While the tumor sizes grew substantially in control mice (Fig. 2b), the majority of PBI-05204 (40 mg/kg) treated mice showed either no visible tumors or reduced tumor size after 6 weeks of treatment (Fig. 2e or h). These findings were further supported by the necropsy of normal appearing pancreas in PBI-05204 treated Panc-1 bearing mice (Fig. 2f) compared to that of control mice (Fig. 2c). In contrast, gemcitabine (40 mg/kg) reduced neither tumor incidence nor growth of this particular tumor (Fig. 1). Mice treated with 125 mg/kg of gemcitabine also failed to show any significant antitumor efficacy (data not shown). When mice were treated with PBI-05204 (20 mg/kg) and gemcitabine together, both tumor weight and tumor burden were significantly reduced compared to that of control group and gemcitabine alone group (*P* = 0.038, *n* = 8 and *P* = 0.013, *n* = 8, respectively) (Fig. 1).

### Anatomical and histological examination of PBI-05205-treated pancreatic tumors

**Fig. 3:**
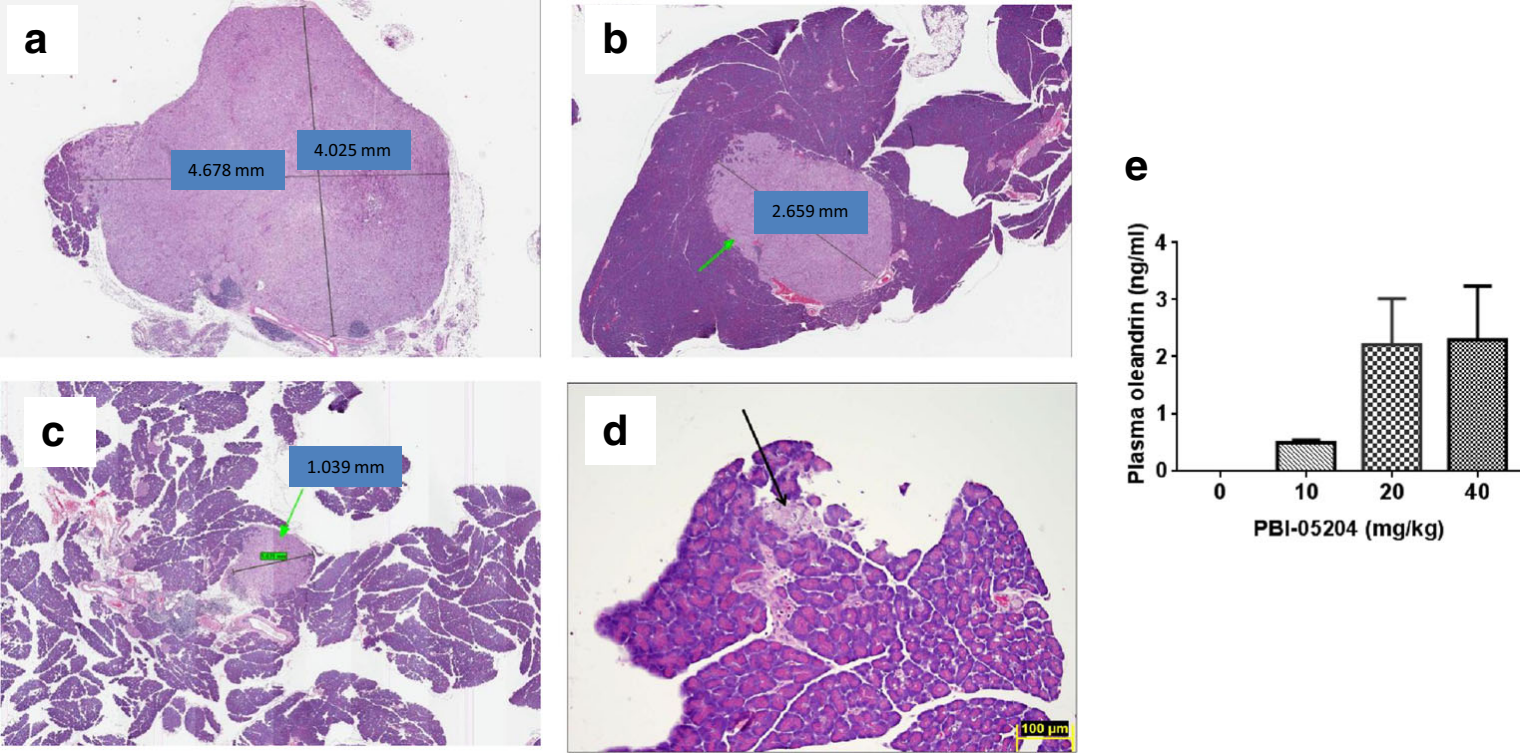
Mouse pancreas bearing orthotopic Panc-1 human tumor, H&E stained sections. **a**. vehicle control; **b**. PBI-05204 (10 mg/kg); **c**. PBI-05204 (20 mg/kg); **d**. PBI-05204 (40 mg/kg). Histological examination of tissue sections showed that PBI-05204 inhibited Panc-1 tumor development in a concentration dependent manner. Pancreatic tumor is significantly smaller in 40 mg/Kg PBI-05204 treated mice (*black arrow* indicating a tumor of less than 100 μm diameter) in comparison with 1.039 mm diameter tumor (*green arrow*) in 20 mg/Kg PBI-05204 treated mice, 2.659 mm diameter tumor (*green arrow*) in 10 mg/Kg PBI-05204 treated mice, and 4.678 mm diameter tumor in control mice. *E. Plasma* concentration of oleandrin in mice treated with vehicle control or PBI-05204. Plasma was collected 4 h after the last dose of PBI-05204 was administered

To further confirm the presence of orthotopic pancreatic tumors, the pancreas with tumor tissue were processed histologically in H&E stained sections that were examined microscopically by a certified pathologist. Pancreases without grossly detectable tumor were serially sectioned along with the mesenterium at 50 μm intervals and stained with H&E for histological examination. Detected pancreatic tumors were analyzed morphometrically. Histological examination of H&E stained tissue sections from PBI-05204 treated (0, 10, 20, 40 mg/kg) mice revealed a dose dependent decrease in tumor size (Fig. 3b–d) compared to that of vehicle control group (Fig. 3a). Intriguingly, histological examination of H&E stained tissue of the serially sectioned pancreas from PBI-05204 (40 mg/kg) treated mice presented with either no detectable tumor cells (5/6) or significantly smaller tumor sizes with diameters less than 2.3 mm (1/6). Given that oleandrin, a major component of PBI-05204, is known to inhibit the proliferation of Panc-1 cells [4], the plasma concentration of oleandrin was analyzed in mice treated with PBI-05204. Figure 3e showed that plasma oleandrin in PBI treated mice increased from an undetectable level (vehicle control) to 2.298 ± 0.945 ng/ml in PBI-05204 (40 mg/kg) treated group collected 4 h after the last dose of PBI-05204.

### PBI-05204 inhibited proliferation of Panc-1 orthotopic tumors and in vitro in the Panc-1 cells

**Fig. 4:**
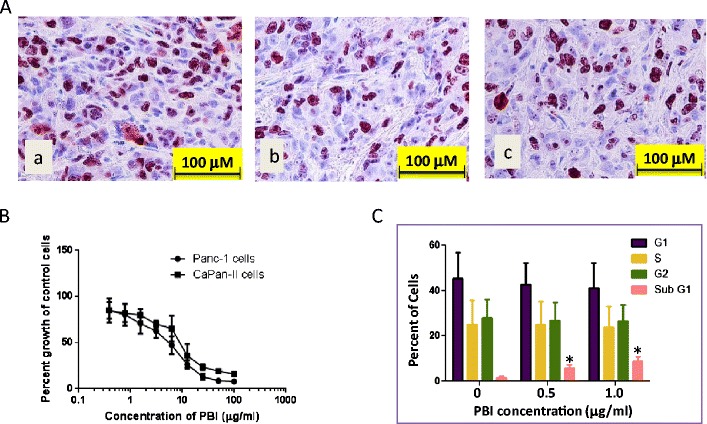
PBI-05204 markedly inhibited tumor proliferation in the Panc-1 orthotopic model as evidenced by a reduction of Ki-67 staining (**A**) and in human pancreatic cancer cells (**B**) by induction of apoptosis (**C**). **A**, *a*). Vehicle control; *b*) PBI-05204 (10 mg/kg); *c*) PBI-05204 (20 mg/kg). **B**. Proliferation of Panc-1 and CaPan-II cells treated with PBI-05204 for 72 h measured by MTT assay. **C**. PI staining of Panc-1 cells treated with PBI-05204 for 48 h. * *P* < 0.05 PBI-05204 treated versus vehicle treated group

To further evaluate the impact of PBI-05205 on cell proliferation of Panc-1 tumors, the relative anti-proliferative capacity in control and PBI-treated mice were examined by Ki-67 staining. As shown in Fig. 4A, Ki-67 staining was markedly reduced by PBI-05204 compared to that in tissues from vehicle treated mice. Similarly, PBI-05204 inhibited the proliferation of Panc-1 and CaPanII human pancreatic cancer cell lines with an IC_50_ of about 10 μg/ml (Fig. 4B). At a 1 μg/ml concentration, PBI-05204 showed about a 5-fold increase in cells at the subG1 phase of the cell cycle, indicative of apoptosis. PBI-05204 treatment led to a concentration dependent reduction of proliferation in Panc-1 cells (Fig. 4C).

### PBI-05204 inhibited PI3kinase/mTOR pathway

**Fig. 5:**
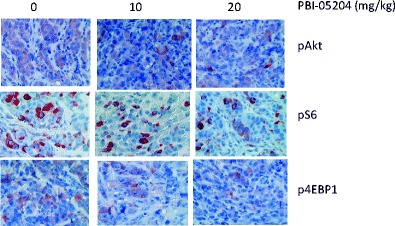
Oral administration of PBI-05204 down regulated the PI3kinase/mTOR pathway proteins in human Panc-1 orthotopic tumors. Tumor sections were obtained by the end of 6 weeks treatment and stained with antibodies raised against pAkt (Ser473), pS6 (Ser 235/236) and p4EBP1 (Thr37/46) proteins and counter stained with hematoxylin

**Fig. 6:**
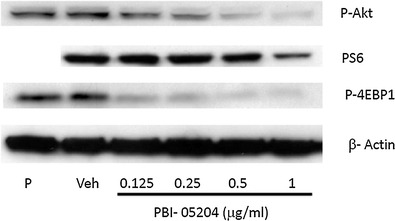
PBI-05204 inhibited the expression of signaling proteins associated with PI3kinase/mTOR pathways in Panc-1 cells. Panc-1 cells were treated with DMSO or PBI-05204 (0.25 to 1 μg/ml) for 24 h. The proteins of interest were measured by western blotting. P. Parental Panc-1 cells without treatment

To understand the molecular mechanisms that are associated with PBI-05204 induced tumor suppressive effects, tumor tissues were subjected to an examination of PI3kinase/mTOR pathways that had previously been reported by us as being involved in the antitumor effect of oleandrin [4]. As shown in Fig. 5, the immunohistochemical staining of phosphorylated Akt, S6 and 4EBP1 in PBI-05204 (10 and 20 mg/kg) treated tumor tissue were all notably reduced compared to that of the control vehicle treated group. When Panc-1 cells were treated with PBI-05204 (0.125 to 1 μg/ml) for 24 h, the expression of these three cell signaling proteins were also down regulated in a concentration dependent manner, suggesting involvement of the PI3K/Akt/mTOR pathways in PBI-05204 elicited antitumor activity (Fig. 6).

## Discussion

In the present study, we investigated the role of a natural product containing oleandrin, a supercritical CO_2_ extract of *Nerium oleander* (PBI-05204), on pancreatic tumor growth in vivo and in vitro. Our results show that PBI-05204 significantly inhibited the peritoneal dissemination of orthotopically implanted Panc-1 cells in nude mice along with inhibition of proliferation and induction of apoptosis. Furthermore, PBI-05204 significantly down-regulated the expression of biomarkers relevant to PI3k/Akt and mTOR pathways in vivo and in vitro, indicating that PBI-05204 targets components of the PI3k/Akt/mTOR pathway to inhibit the growth of primary tumors. Given that a recent study using patient derived human pancreatic cancer xenograft models and their primary tumor suggests that cardiac glycosides, including digoxin, digitoxin and ouabain, inhibit the proliferation of both primary cancer cells and their xenograft tissues [18], our study for the first time demonstrates a natural product containing oleandrin that has already completed a successful clinical Phase I safety trial has the ability to strongly inhibit the growth of human pancreatic cancer in an orthotopic model.

Cardiac glycosides, such as digoxin or digitoxin, are mainly used for the treatment of congestive heart failure. The initial observation of the potential impact of cardiac glycosides on cancer development occurred in 1979, when Dr. Stenkvist’s studies suggested that breast cancer patients who were on digoxin for treatment of congestive heart failure showed a more benign phenotype of their malignant disease than those patients not receiving cardiac glycoside therapy [19, 20]. Since this initial clinical observation, there has been an increased interest in the effect of cardiac glycosides on cancer growth. A large body of literature now indicates that various cardiac glycosides, including both cardienolides (digoxin, digitoxin, oleandrin) and bufadienolides (bufalin, cinobufagin and cinobufotalin), exert important anticancer activity in various human tumor cell and xenograft models [21–26]. Most recently, a study further supports cardiac glycoside mediated anticancer activity in human pancreatic cancer. Authors showed that among over 2000 compounds that had been screened, three cardiac glycosides, digoxin, digitoxin and ouabain, had strong anticancer activity against both primary human pancreatic cancers and their xenograft models [18]. Additionally, studies have also documented that bufalin inhibits human tumor mouse orthotopic models of human hepatocellular carcinoma while UNBS1450 inhibited the growth of glioblastoma [27, 28]. We and others have previously demonstrated that oleandrin inhibits the proliferation of human pancreatic cancer cells, Panc-1 and MiaPaca, human colon cancer cells, melanoma and non-small cell lung cancer cells by either induction of apoptosis or autophagic cell death [4, 7, 16]. In light of the anti-proliferative activity of oleandrin and the supercritical CO_2_ extract of *Nerium oleander* in human pancreatic cancer Panc-1 cells being very similar when they are normalized to relative oleandrin concentrations (data not shown), we examined the antitumor efficacy of PBI-05204, a supercritical CO_2_ extract of *Nerium oleander*, in human pancreatic cancer Panc-1 orthotopic models and have shown that PBI-05204 not only dose dependently slows the growth of this particular tumor, it almost totally eradicates tumor growth at the highest dose (40 mg/kg) after 6 weeks of treatment. In comparison, the standard therapeutic agent gemcitabine (at either 40 or 125 mg/kg) failed to suppress the proliferation of this particular orthotopic tumor model. Intriguingly, the plasma oleandrin levels in PBI-05204 (40 mg/kg) treated mice were previously found to be achievable in humans without any cardiac toxicity based on the result of a phase I trial of PBI-05204 [29].

Pancreatic ductal adenocarcinoma (PDA) remains one of the most aggressive tumors in humans with a 5-year survival rate of less than 5 %[30]. The standard systemic chemotherapy for PDA is gemcitabine following its approval for this disease in 1997. However, treatment with gemcitabine results in only a marginal increase in pancreatic cancer patient survival. Because of this poor response, many efforts have been focused on improving the therapeutic potential of gemcitabine in PDA through a combination of radiation, chemotherapy as well as targeted therapeutic agents [31–34]. Among more than a dozen combination therapy trials, only a few, such as gemcitabine and Abraxane, have shown any improved survival benefit compared to gemcitabine alone. Clearly, new targets or therapeutic approaches are needed for this disease. In our model, gemcitabine alone failed to demonstrate any tumor suppressive effect, which was consistent with results of previous studies [35]. When mice with Panc-1 orthotopic tumors were treated with a combination of PBI-05204 (20 mg/kg) with gemcitabine (40 mg/kg), tumor growth was significantly and substantially slower compared to mice treated with vehicle control or gemcitabine alone. The reduction of tumor growth in gemcitabine and PBI-05204 treated mice was also stronger than that of PBI-05204 (20 mg/kg) treated mice.

One of the limitations of this study is that the Panc-1 xenograft model responded poorly to gemcitabine treatment alone which may have been due to the lack of expression of S100P protein [35]. To be useful clinically, further studies will be needed to determine whether PBI-05204 can potentiate the therapeutic effect of gemcitabine as well as other approved chemotherapeutic agents in human pancreatic cancer orthotopic models that are relatively responsive to treatment of gemcitabine, such as MiaPaca and AsPC-1 cells. Such studies are currently underway.

While a large body of evidence suggests that cardiac glycosides exert anticancer activity against various cancers including both solid and non-solid malignant diseases, numerous molecular mechanisms responsible for the antitumoral effect of CGs have been proposed [24]. Suggested targets include NF-*k*B, ERK and PI3kinase pathways. We have previously reported that oleandrin down regulates phosphorylation of Akt in Panc-1 cells, and that addition of activated Akt abrogates the oleandrin elicited anticancer activity in these particular cells [7]. Similarly, dephosphorylation of Akt or inhibition of Akt phosphorylation was observed in U937 cells or TPA-induced activation of PI3kinase/Akt in mouse skin treated with oleandrin, respectively [36, 37]. Additionally, Zhang D. *et al.* demonstrated that aerobufalin, a bioactive component of bufo toad, induces apoptosis by inhibiting both PI3kinase/mTOR pathways in human hepatocellular carcinoma HepG2 cells [38]. Notably, in this current study, PBI-05204 treatment led to down-regulation of both PI3kinase/mTOR pathways as evidenced by reduced expression of pAKT, pS6 and p4EBP1 in both Panc-1 cells and its relevant orthotropic model. This provides scientific support to the results of the phase I trial of PBI-05204 which also showed that both PI3kinase/mTOR signaling pathways were down regulated in PBMCs of some patients enrolled in the study [26]. In fact, the down regulation of pS6 appeared to be more pronounced than that of the reduction of pAkt in both the Panc-1 orthotopic model and PBMCs of phase I patient samples [29], which suggests that the down regulation of the mTOR pathway is important in PBI-05204 elicited antitumor activities. In addition to PDA, emerging evidence supports that PI3kinase/mTOR pathways are critical in pancreatic neuroendocrine tumors (PanNETs) due to PTEN deletion, tuberous sclerosis complex mutation and activation of Akt and mTOR [39]. Studies of the impact of PBI-05204 in human PanNETs may be interesting and informative.

In conclusion, we have used an orthotopic model of human pancreatic Panc-1 cancer to identify the antitumor efficacy of PBI-05024 and its relevant molecular mechanisms. Compared to gemcitabine, the commonly used chemotherapeutic agent for PDA, PBI-05204 exhibited strong monotherapeutic antineoplastic effects through down regulation of PI3k/mTOR pathways and augmentation of the antitumor efficacy of gemcitabine. Given that the phase I clinical trial of PBI-05204 showed very limited cardiac toxicity, these data provide further support for PBI-05204 as a potential anticancer therapeutic agent for pancreatic cancer that warrants further clinical investigation.
